# Development and Quality Enhancement of Fried Fish Cake Prototype with Transglutaminase, Trehalose, and Herbal Oil for Room Temperature Distribution

**DOI:** 10.3390/gels10110740

**Published:** 2024-11-14

**Authors:** Ye Youl Kim, Khawaja Muhammad Imran Bashir, Seonyeong Kang, Yongjoon Park, Jae Hak Sohn, Jae-Suk Choi

**Affiliations:** 1 Samjin Food Co., Ltd., Busan 49036, Republic of Korea; dudndi1324@samjinfood.com (Y.Y.K.); glorysun@samjinfood.com (S.K.); sj0001@samjinfood.com (Y.P.); 2 Department of Seafood Science and Technology, Institute of Marine Industry, Gyeongsang National University, Tongyeong 53064, Republic of Korea; imranbashir@gnu.ac.kr; 3 German Engineering Research and Development Center for Life Science Technologies in Medicine and Environment, Busan 46742, Republic of Korea; 4 Department of Food Science and Culinary Arts, College of Health and Welfare, Silla University, Busan 46958, Republic of Korea; 5 Seafood Research Center, IACF, Silla University, 606, Advanced Seafood Processing Complex, Busan 49277, Republic of Korea

**Keywords:** gel strength, high-pressure treatment, high-temperature processing, product enhancement, response surface analysis, storage duration, surimi-derived products

## Abstract

This study focuses on developing a fried fish cake prototype with improved quality and extended shelf-life, enabling room-temperature distribution through an innovative high-temperature and high-process retort method. Surimi-based products typically necessitate cold storage and a refrigerated distribution system, affecting their physical properties and flavor while escalating costs. By incorporating Transglutaminase (TGase), trehalose, and herbal oils, and optimizing the heating process using the response surface methodology, this research addresses challenges related to changes in physical properties, color, and off-flavors during high-temperature and high-pressure treatment. The addition of 0.37% ACTIVA-K TGase significantly enhanced gel strength by promoting protein cross-linking, while 0.75% trehalose improved color stability by suppressing browning, thus enhancing visual appeal. A 0.1% concentration of bay oil effectively enhanced the flavor profile by masking undesirable odors without compromising the sensory quality. Optimized processing conditions maximized DPPH radical scavenging activity, whiteness, and gel strength, ensuring superior product quality and safety. Nutritional analysis confirmed a balanced composition of moisture, protein, essential amino acids, and minerals, in accordance with Korean national standards for acid values. Microstructural examination revealed a uniform network structure, contributing to excellent texture and sensory evaluations. Shelf-life predictions indicated a storage duration of approximately 19 months, surpassing commercially available products and offering a competitive edge. This novel approach allows surimi-based products to be stored and distributed at room temperature, while also providing the potential for increased profitability.

## 1. Introduction

In the global food industry, surimi—a refined concentrate of myofibrillar proteins—is lauded for its nutritional benefits, primarily its high protein and low-fat content. Derived from fish meat through the removal of sarcoplasmic proteins, blood, fat, and pigments, surimi forms the foundation for a variety of health-oriented food products [1]. Fish cakes, among the myriads of surimi products, are particularly popular due to their versatility in preparation methods like steaming, baking, and frying. In Korea, frying is preferred for its ability to enhance the visual appeal, flavor, and texture of fish cakes, as well as its efficiency in reducing cooking time [2].

As of 2022, Korea’s domestic production of fried fish cakes was approximately 100,000 tons, accounting for 56% of all surimi products. These products are primarily distributed under refrigeration, offering a shelf-life of up to 10 days for unsterilized and up to 50 days for sterilized varieties. The export volume, ranging between 9814 and 11,100 tons, is expected to grow [3]. However, frozen exports face quality challenges such as sponginess and drip loss upon thawing, which restricts market expansion both domestically and internationally.

To facilitate room temperature distribution, effective sterilization processes are crucial. Traditional methods like high temperature and high-pressure treatments are favored for their safety and convenience, yet they can lead to nutrient degradation, texture deterioration, and undesirable sensory effects such as off-flavors or discoloration [4]. This has spurred research into enhancing texture with additives like transglutaminase (TGase) [5,6], konjac [7,8], and carrageenan [9,10].

TGase is a promising agent for improving the quality of meat products by enhancing gel strength through the formation of ε-(γ-glutamyl)-lysine cross-links [11,12,13,14]. It is particularly effective in surimi products, promoting cross-linking and protein aggregation at elevated temperatures [4,13]. It has been reported to enhance the gel strength of surimi products [15]. While at low temperatures, the cross-linked protein structure inhibits hydrogen bond formation; at high temperatures, it promotes cross-linking and aggregation of surimi proteins [4,13].

The Maillard reaction leads to alterations in protein digestibility, protein functionality, and the sensory characteristics of food, which in turn affect the color of the food [16]. Therefore, it is crucial to manage Maillard reaction during food processing, particularly during production and storage, to preserve the desired quality of the food. In high-pressure processing, sugars like glucose, sucrose, and trehalose are incorporated into food products to prevent discoloration and extend shelf-life [16,17]. Trehalose is particularly favored in food processing due to its superior stabilizing properties [18,19,20]. Trehalose is a white, odorless powder with a sweetness approximately 45% that of sucrose. It is a non-reducing disaccharide comprising two α-glucose molecules valued for its ability to prevent starch aging and stabilize proteins and lipids [21]. It is naturally found in algae, yeast, fungi, plants, and insects [22]. Its applications span food processing, cosmetics, and pharmaceuticals, with research emphasizing its benefits in inhibiting starch aging [23,24], enhancing water holding capacity [25] and storage properties in agricultural product processing [24]. Despite its versatility, limited studies exist on its effectiveness in preventing color changes in processed fishery products like fish cakes [4]. Additionally, the incorporation of herbal oils, known for their antimicrobial and antioxidant properties, presents a novel approach to enhancing the sensory attributes and extending the shelf-life of food products.

This research explores the synergistic effects of TGase, trehalose, and herbal oils in developing a high-quality fried fish cake prototype suitable for room temperature distribution. The combination of TGase, trehalose, and herbal oils was selected based on our preliminary study of different combinations and the recent report by Lee et al. [4]. The study aims to optimize processing conditions that maintain product integrity during high-temperature and high-pressure treatment, thereby supporting the expansion of fish cake markets globally. Furthermore, the physicochemical and sensory quality characteristics of the newly developed prototypes, along with their extended shelf-life, are thoroughly evaluated to ensure a competitive edge in both domestic and international markets.

## 2. Results and Discussion

### 2.1. Addition of TGase and Sugars in Fried Fish Cake Prototype

#### 2.1.1. Gel Strength Improving Effect of TGase

To evaluate the texture related to heating and TGase addition, the gel strength of the fried fish paste prototype was measured, and the results are shown in Table 1. The test product that was heat-treated with added TGase showed the highest gel strength value of 156.52 g × cm. TGase is an enzyme that forms an ε-(γ-Glu)Lys bond by inducing a cross-link between the γ-carboxyamide group of the glutaminyl residue and the ε-amino group of the lysine residue. When used in food, this enzyme is known to form cross-links between proteins, thereby improving gel formation, coagulation, heat stability, hydration, solubility, and adhesiveness of the food [26,27]. This could explain the higher gel strength observed in the samples containing TGase.

Numerous studies have reported improvements in the textural properties of surimi and surimi-based products through the application of various protein additives [28,29,30,31]. In our study, we utilized ACTIVA TG-K to improve the texture of a newly developed fried fish cake prototype. This additive facilitates deamidation, amine incorporation, and protein cross-linking [32]. By forming ε-(γ-glutamyl) lysine bonds within the myosin heavy chain during the setting of salted fish paste, ACTIVA TG-K enhances texture, leading to a firmer and more cohesive consistency [33]. In line with previous research on the use of transglutaminases to enhance gel strength [20,32,33,34,35], our results showed a significant increase in gel strength in the fried fish cake prototype with 0.37% ACTIVA TG-K.

#### 2.1.2. Gel Strength and Color Improvement Effect of Added Sugars

To examine the texture and color, the addition of TGase and the heating process (55 °C, 10 min) were applied in the same manner. When the gel strength, yellowness, and whiteness of the samples with different sugars added were measured, the sample with added trehalose showed the highest gel strength at 187.34 g × cm (Table 2). The elevated gel strength is attributed to trehalose’s ability to maintain an active form, which stabilizes protein molecules [36].

In examining the color change in the sample products based on the type of sugar, in terms of yellowness, the sample product with added xylose showed the highest value at 26.05, while the sample with added trehalose was the lowest at 6.53. Regarding whiteness, the product with added trehalose was the highest at 49.29, and the product with added xylose added was the lowest at −31.06 (Table 2).

When heat is applied to a mixture of fish cake dough, fish meat surimi, and sugar, the Maillard reaction occurs as the amino group of the amino acids combines with the carbonyl group of the reducing sugars. Xylose, a 5-carbon sugar, has a more reactive reducing group than other sugars, leading to a more pronounced yellowness [37]. Trehalose, a naturally occurring disaccharide composed of two glucose molecules linked by an α-1,1 glycosidic bond, is a non-reducing sugar known for its chemical stability and resistant to acids, alkalis, and the Maillard reaction. This makes it a popular choice in various food applications [38]. In this study, we observed that browning was minimized when heating the fish cake prototype with added trehalose. The improved gel strength in the fried fish cake prototype containing trehalose can be attributed to its capacity to stabilize protein molecules, thereby enhancing the overall gel structure in the fish cakes [21,36].

#### 2.1.3. Flavor Improvement by Addition of Herbal Oil

Defrosted fish cake products often experience nutrient degradation, texture loss, changes in physicochemical properties, taste alterations, and the development of off-flavors or discoloration [39]. To address these issues, we enhanced the fish cakes with various herbal oils. The results for odor intensity and sensory evaluation of the test products with added tocopherol and various herbal oils are presented in Table 3. The fried fish cake with bay oil demonstrated a particularly suitable odor intensity (468.8 ± 22.1) and received higher scores in sensory evaluations (7.47 ± 0.2). Although the fried fish cake prototype supplemented with tocopherol had a slightly higher sensory evaluation score than the one with bay leaf oil and a lower odor intensity (388.6 ± 19.4), the fragrance was not preferred by sensory evaluators. Consequently, bay oil, which had a relatively lower odor intensity and high sensory evaluation scores, was identified as the most suitable herb oil for the fried fish cake prototype.

### 2.2. Optimal Processing Conditions for Fried Fish Cake Prototype

The DPPH (*Y*_1_), a dependent variable of the fried fish cake sample with added TGase, laurel oil, and trehalose, tended to increase as *X*_1_ (amount of antioxidant added) moved from −1.682 to +1.682. For both *X*_2_ (temperature conditions during high-temperature and high-pressure treatment) and *X*_3_ (time conditions for high-temperature and high-pressure treatment), DPPH (*Y*_1_) value showed a decreasing trend as it shifted from −1.682 to +1.682. Another dependent variable, whiteness (*Y*_2_), did not change much as *X*_1_ moved from −1.682 to +1.682. However, as *X*_2_ and *X*_3_ moved from −1.682 to +1.682, the whiteness (*Y*_2_) of the prototype tended to decrease.

**Figure 1:**
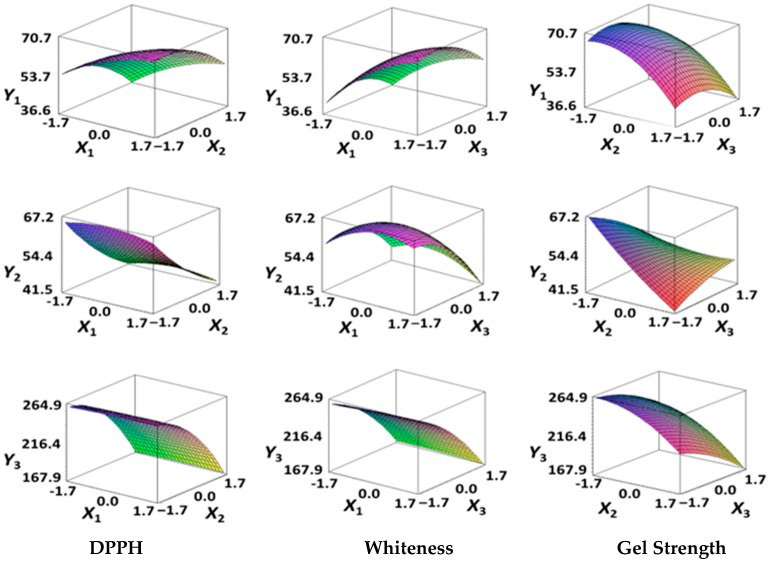
Three-dimensional response surface graph illustrating the correlation between independent variables (X1, X2, and X3) and dependent variables (Y1, Y2, and Y3). X1: Amount of antioxidant added (%); X2: Temperature conditions during the high-temperature and high-pressure treatment process (°C); X3: Time conditions in the high-temperature and high-pressure treatment process (min); Y1: DPPH (%); Y2: Whiteness; Y3: Gel strength (g × cm).

This indicates that the amount of antioxidant added does not significantly affect the whiteness of the prototype, whereas the temperature and time during the high-temperature and high-pressure treatment process have a significant impact. In the case of *X*_1_, there was no significant change in the gel strength (*Y*_3_) of the prototype as it moved from −1.682 to +1.682. Conversely, for *X*_2_ and *X*_3_, there was a tendency for the gel strength (*Y*_3_) of the prototype to decrease as it moved from −1.682 to +1.682. It is also believed that gel strength is not affected by antioxidants but is influenced by the conditions of high-temperature and high-pressure treatment (Figure 1).

The lack of fit test, used to assess the suitability of the response model equation for the dependent variable, yielded *p* values of 0.095, 0.083, and 0.073 for DPPH (*Y*_1_), whiteness (*Y*_2_), and gel strength (*Y*_3_), respectively, all of which are close to 1, indicating that the design model was appropriate [40] (Table 4).

Conditions involving high-temperature and high-pressure treatment of fried fish cake prototypes enhance safety through complete sterilization when treated at higher temperatures and for longer periods. However, excessive treatment may cause the deterioration of the quality of the fish cake. Additionally, the antioxidant effect may decrease due to heat and pressure. Considering these aspects, an appropriate time and temperature range is needed for manufacturing a high-quality and microbiologically safe fried fish cake prototype. Consequently, the desired values of the dependent variables [DPPH (*Y*_1_), whiteness (*Y*_2_), and gel strength (*Y*_3_)] for determining optimal high-temperature and high-pressure treatment conditions were set to a maximum value of 70% for DPPH, 53.0 for whiteness, and 230.0 g × cm for gel strength based on the experimental results (Table 5).

The optimal amount of antioxidant added and the sign values for high-temperature and high-pressure treatment temperature and time that satisfied all dependent variables, considering the target values of independent variables for the fried fish cake prototype, were 1.08, −0.49, and 0.75, respectively, with actual values of 0.15%, 112.6 °C, and 28.8 min (Table 6).

### 2.3. Nutritional Properties of Fried Fish Cake Prototype

#### 2.3.1. Proximate Composition, pH, and Energy

The developed fried fish cake prototype had the highest moisture content at 72.4 g/100 g, which was similar to that of control group 3 but significantly different from control groups 1 and 2 (Table 7). In terms of protein, control groups 1 and 2 had a slightly higher content than the developed fried fish cake prototype, likely because the prototype had a high moisture content and a relatively low protein content (10.3 g/100 g). There was no significant difference in fat content among all products. Control group 2 had the highest ash content, while control groups 1 and 2 had the highest carbohydrate content. There was no significant difference in pH among all samples. Control groups 1 and 2, with a high carbohydrate content, showed high energy values.

#### 2.3.2. Total Amino Acid

The total amino acid content of the fried fish cake prototype was 10.17 g/100 g, with essential amino acids constituting 5.07 g/100 g (49.9%). The major amino acids (more than 8% of the total amino acids) in the fried fish cake prototype were aspartic acid (10.7%), glutamic acid (16.3%), leucine (8.9%), and lysine (9.4%) (Table 8). The variation in amino acid content between the fried fish cake prototype and the three control groups was attributed to variations in protein content resulting from the mixing conditions, while the proportions of each amino acid remained largely consistent.

#### 2.3.3. Minerals

The mineral composition of surimi and fish cakes is an essential criterion for surimi products and has a direct impact on consumer preferences [42]. The calcium content of the developed fried fish cake prototype was 35.3 mg/100 g, phosphorus was 116.0 mg/100 g, potassium was 66.4 mg/100 g, iron was 0.05 mg/100 g, and magnesium was 14.1 mg/100 g (Table 9). The mineral content of the control groups and the original product was measured, revealing differences in the mineral content of the fish cake samples depending on the type of surimi used as the main ingredient. This trend was consistent with the findings of [43], who observed that golden-threadfin bream surimi had a higher content of all minerals compared to pollack surimi, presumably due to the intrinsic mineral content differences in the surimi itself [43].

#### 2.3.4. Fatty Acids

The total fatty acid content of the fried fish cake prototype was 1.03 g/100 g, which constituted 73.6% of the crude fat content (Table 10). In the samples analyzed, linoleic acid was found in significantly lower amounts in the fried fish cake prototype compared to the control groups. Margaric acid, arachidic acid, behenic acid, γ-linolenic acid, and erucic acid were present in small quantities in the control samples but were absent in the fried fish cake prototype. Conversely, elaidic acid was detected only in the fried fish cake prototype in trace amounts, not in the controls. These differences in fatty acid composition between the prototype and the controls are likely due to variations in manufacturing composition and processing conditions. Typically, during fish cake production, the tender fish meat is washed to remove contaminants such as lipids, pigments, and blood, resulting in low lipid content. The crude fat and fatty acids found in both the fried fish cake prototype and the control groups are attributed to the soybean oil used in the frying process.

#### 2.3.5. Odor Intensity and Volatile Basic Nitrogen (VBN)

The odor intensity of the developed fried fish cake prototype was 544.1 ± 14.35, with control group 2 having the highest intensity at 969.30 ± 7.56 and control group 3 the lowest at 535.40 ± 6.26. The VBN content of the developed prototype was 1.1 mg/100 g, with control group 2 being the highest at 3.1 mg/100 g, and control group 3 the lowest at 1.0 mg/100 g, following the same trend as odor intensity (Table 11). It was determined that the fishy odor of threadtail sea bream surimi used in control groups 1 and 2 was more pronounced, affecting both the odor intensity and VBN content. The increased odor intensity in control group 2 compared to control group 1 and the developed prototype suggests that heat treatment increased substances contributing to odor intensity. Despite heat treatment, the fishy smell was masked by the herbal oil addition, resulting in high sensory evaluation scores for flavor in both control group 3 and the developed prototype, which was consistent with overall preference trends.

#### 2.3.6. Acid Value

The acid value was not detected in the developed fried fish cake prototype at 0.01 mg/g (Table 11). This was attributed to the very low crude fat content and the prevention of oxidation through vacuum packing and subsequent heating. The Korean Standards (KS standards) for acid value in fried fish cake is 2.5 mg/g [44], and the developed prototype meets this standard.

#### 2.3.7. Hunter Color Values

In the Hunter color values of the developed fried fish cake prototype, the L value was the highest at 76.47, with a and b values of −2.29 and 7.55, respectively, and whiteness at 53.83 (Table 11). This result was due to the absence of a reduction reaction of the added sugar, trehalose. The color preference of the developed prototype was the highest at 6.8 points, similar to control group 3 at 6.7 points, aligning with the overall preference trend. Generally, high whiteness is considered a marker of superior product quality [45]. Therefore, the addition of trehalose and optimized heating processes were used to minimize yellowness and enhance whiteness based on sensory evaluation results.

Control sample 2 showed a high b value, indicating increased yellowness, likely due to the Maillard reaction occurring during manufacturing, which involves a roasting stage. The ΔE value for the newly developed fried fish cake prototype indicated only slight color variation, attributed to the lower b values (yellowness) resulting from the addition of trehalose. Typically, fish cake products with higher whiteness are perceived as superior [46]. The increased whiteness in the fried fish cake prototype can be attributed to the incorporation of trehalose and optimized processing conditions. This prototype exhibited approximately 40% greater whiteness compared to control sample 1, however there was no significant difference when compared to control 3. Control sample 3 was produced under similar conditions but did not undergo sterilization. As a result, even though control 3 showed high whiteness, it was not significantly different from that of the developed fried fish cake prototype.

#### 2.3.8. Gel Strength

The fried fish cake prototype demonstrated the highest gel strength, measuring 279.3 ± 15.42 g × cm (Table 11). Gel strength serves as a crucial quality indicator for fish cakes, where a higher breaking force suggests a firmer texture, and a greater penetration depth indicates higher elasticity. Therefore, a fish cake with significant penetration depth is considered to be of high quality.

Gel strength is a vital textural attribute that subsequently affects the quality and pricing of fish cake products [28,29,47]. It is calculated by multiplying the indentation strength (g) by the depth (cm), where indentation represents the applied force, and depth refers to the distance until this force peaks [48]. Generally, greater indentation strength indicates firmer physical properties, while a higher depth suggests that the force decreases gradually, reflecting high elasticity. The average indentation strength and depth values for the newly developed fried fish cake prototype and control groups 1, 2, and 3 were 331.14, 458.80, 305.50, and 362.67 g, and 0.70, 0.56, 0.80, and 0.77 cm, respectively. The developed fried fish cake prototype exhibited the highest gel strength among the samples, characterized by its highly elastic fish paste form and substantial depth.

### 2.4. Gel Electrophoresis

**Figure 2:**
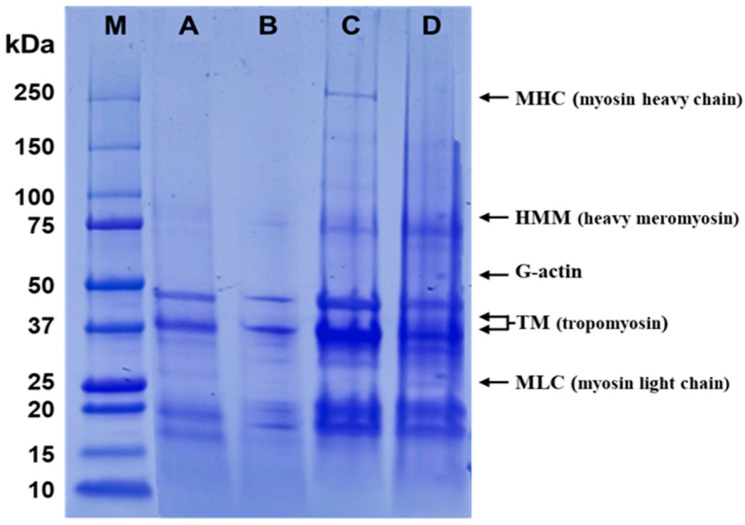
Protein distribution pattern in the developed fried fish cake prototype and the controls observed by SDS-PAGE. M: Molecular weight marker; A: Control 1; B: Control 2; C: Control 3; D: Developed fried fish cake prototype.

The SDS-PAGE analysis results of the developed fried fish cake prototype and control groups showed the myosin light chain of the prototype (D) and control group 3 (C) was most clearly identified in the 15–20 kDa range, followed by control group 2 (B) and control group 1 (A) (Figure 2). In the 20–25 kDa range, α-tropomyosin of the prototype (D) was identified. In the 25–37 kDa range, β-tropomyosin was most clearly identified in the prototype (D) and control 3 (C), followed by control 1 (A) and control 2 (B). In the 50–75 kDa range, G-actin was observed only in the prototype (D) and control 3 (C). In the 75–100 kDa range, heavy meromyosin was observed in control 3 (C). Among the peptide bands, the largest number appeared in the 37–50 kDa range, with various molecular weights confirmed overall in control 3 (C).

### 2.5. Microstructural Properties

**Figure 3:**
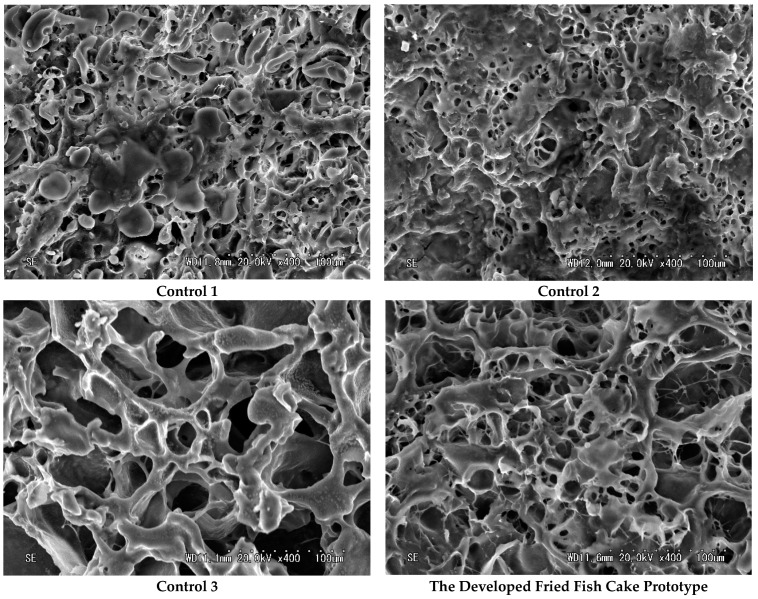
Scanning electron microscope results of controls and the developed fried fish cake prototype (Photo magnification: ×400).

The results of comparing the cross-sections of the developed fried fish cake prototype and control groups using scanning electron microscopy are shown in Figure 3. Control groups 1 and 2 exhibited very small pores and a clumped structure. Smaller pores indicate a weaker water retention capacity, affecting product elasticity. In contrast, control group 3 and the developed prototype had larger pores and a dense network structure, suggesting a better water retention capacity. This structural difference is presumed to be due to the presence or absence of the TGase enzyme, which maintains the protein network structure, and differences between threadtail sea bream meat (control groups 1 and 2) and pollack meat (control group 3 and the prototype).

The physical properties of the fish cake showed little difference in gel strength based on meat type and high-temperature and high-pressure treatment. However, microstructural observation confirmed that the regular network structure became less uniform, irregular, and clumped due to high-temperature and high-pressure treatment. Gel strength is the product of breaking force and penetration distance. In this experiment, no significant difference in gel strength was observed between the controls and the prototype. However, sensory evaluation showed the prototype with a uniform network structure had a superior texture and overall preference. This suggests that when subjected to high-temperature and high-pressure treatment, the added food additives, TGase and trehalose, prevented uneven protein network structure deterioration, ultimately increasing prototype preference.

### 2.6. Sensory Evaluation

The sensory evaluation results of the fried fish cake sample are presented in Table 12. The taste was rated at 6.8 ± 0.22 points, color at 6.6 ± 0.13 points, aroma at 6.3 ± 0.16 points, texture at 6.7 ± 0.11 points, and overall preference at 6.7 ± 0.15 points—all higher than control groups 1 and 2, and similar to control group 3.

The newly developed fried fish paste cake prototype serves as a basic fish cake product, as it does not contain any additional auxiliary ingredients with strong flavor profiles such as vegetables or cheese. This allows for the customization of flavor by adding preferred auxiliary ingredients, even those with intense taste characteristics. The odor rating for this prototype was 6.3 ± 0.16, which was higher than that of grilled mackerel, grilled Spanish mackerel, grilled flounder, canned sardines, vegetable fish paste cake, and fish paste cake bars. In contrast, the control samples had a fishy smell, likely due to differences in frying, sterilization, and storage processes. Consequently, it was determined that the developed fried fish cake prototype possesses the most suitable odor score in comparison to the control samples. The prototype’s appearance (color) was almost identical to the white color in the Munsell Table. Trehalose played a significant role in enhancing whiteness, indicating its usefulness in reducing color changes. The texture (elasticity) score of the prototype was 6.7 ± 0.11 points, surpassing that of acorn jelly, vegetable fish paste cake, konjac, and fish paste cake bars. This favorable result is due to the inclusion of TGase and the optimization of processing conditions involving high-temperature and high-pressure.

### 2.7. Shelf-Life Prediction

During 150 days of storage, the total bacterial counts (TBC), *Escherichia coli*, and coliform group, which are important indicators of food hygiene and microbiological characteristics according to storage temperature (25 °C, 35 °C, 45 °C), were not detected, indicating no bacterial presence at any storage temperature or period. *E. coli* and coliform bacteria were negative regardless of storage temperature and period.

The acid value and sensory changes, indicating fat oxidation degree according to storage temperature, are shown in Table 13. During storage, the acid value of the prototype ranged from 1.2 to 6.6 mg KOH/g at 25 °C, 1.2 to 10.2 mg KOH/g at 35 °C, and 1.2 to 12.6 mg KOH/g at 45 °C, all increasing with storage duration. Herbs are known to inhibit fat oxidation during food processing and storage [49]. The overall preference of sensory evaluation was measured using a 7-point scale, ranging from 6.1 to 7.0 points at 25 °C, 5.5 to 7.0 points at 35 °C, and 5.2 to 7.0 points at 45 °C, all decreasing over the storage period.

The expected shelf-life of the developed fried fish cake prototype is shown in Table 13. Park et al. [50] described that a model with a high *R*^2^ value is suitable for predicting shelf-life. In this study, the predicted shelf-life (23.93 months) was determined using the 0th model results, showing a higher *R*^2^ than the 1st model at each storage temperature. However, multiple factors influence shelf-life conditions independent of how the product is handled during production, distribution, and storage [45]. Therefore, the final shelf-life was determined to be approximately 19 months after applying a safety factor of 0.8. This is approximately 11 times longer than the commercially available control group 1, which has a shelf-life of approximately 50 days (Table 14).

## 3. Conclusions

This research successfully developed a fried fish cake prototype with enhanced quality attributes and an extended shelf-life, suitable for room-temperature distribution. By integrating TGase, trehalose, and bay oil, and optimizing the heating process via the response surface methodology, the study effectively addressed the challenges associated with high-temperature and high-pressure treatments. TGase played a crucial role in enhancing gel strength through protein cross-linking, while trehalose improved color stability by reducing browning, thereby increasing the product’s visual appeal. Bay oil was instrumental in enhancing the flavor profile by effectively masking undesirable odors, ensuring a pleasant sensory experience. The optimized processing conditions not only maximized antioxidant activity and maintained desirable whiteness and gel strength but also ensured compliance with nutritional standards, including a balanced composition of moisture, protein, essential amino acids, and minerals. The uniform microstructure observed in the prototype contributed to its superior texture and sensory evaluations. Furthermore, the shelf-life prediction indicated a storage duration of approximately 19 months, offering a competitive edge over existing products. This innovative approach not only facilitates room-temperature storage and distribution but also paves the way for broader applications of these techniques in surimi-based product development, potentially increasing profitability and market reach.

## 4. Materials and Methods

### 4.1. Materials

The development of the room-temperature fried fish cake prototype utilized the following materials: Alaskan pollock (*Gadus chalcogrammus*) surimi (AA grade; Westward Seafood Inc., Bellevue, WA, USA), transglutaminase (ACTIVA TGase-K, Ajinomoto, Tokyo, Japan), trehalose (Tongliao Meihua Biological Technology Co., Ltd., Langfang, China), D-xylose (Healtang Biotech Co., Ltd., Jinan, China), glucose (Q 1 Co., Ltd., Seoul, Republic of Korea), sucrose (Cheil Jedang Co., Ltd., Seoul, Republic of Korea), herb oils [bay tree (Laurel Noble Oil, Dotterline Co., Ltd., Hwaseong, Republic of Korea), aniseed, coriander, cumin, basil oil (Moellhausen Co., Vimercate, MB, Italy)], tocopherol (mixed tocopherol 70%; Namyung Co., Ltd., Seoul, Republic of Korea), ice (Handong Co., Ltd., Busan, Republic of Korea), refined salt (Solar Salt Co., Ltd., Gwangju, Republic of Korea), fish meat extract (Jin-sung FM Co., Hwaseong, Republic of Korea), monosodium glutamate (Miwo, Daesang Co., Ltd., Seoul, Republic of Korea), glycine (Hebei Huayang Biological Technology Co., Ltd., Hengshui, China), egg white powder (Eurovo SRL Co., Ltd., Emilia-Romagna, Italy), phosphate complex (Polymix, Seodo BNI Co., Ltd., Hwaseong, Republic of Korea), tapioca starch (Moafood Co., Ltd., Gwangju, Republic of Korea), and potato starch (Dongafood Co., Ltd., Ulsan, Republic of Korea).

### 4.2. Manufacturing of Fried Fish Cake Prototype

The mixing ratio of main and auxiliary ingredients for the fried fish cake prototypes followed the guidelines outlined by Lee et al. [4], as detailed in Table 1. In brief, the dough was prepared by mixing the main and auxiliary ingredients, then steamed in a steamer machine (CHDC–500; Chungha Machinery Co., Ltd., Busan, Republic of Korea) at 47 ± 1 °C for 10 min. Subsequently, the steamed fish cake was fried in a fryer (WS-EF S20; Woosung Co., Ltd., Seoul, Republic of Korea) at 160 ± 3 °C for 6 min and cooled to room temperature for 10 min. The cooled fried fish cake was vacuum-packed in a retort pouch (PET12/AL9/NY15/CPP80) using a vacuum packer (TPS-V75; MAPPACK Co., Hwaseong-si, Republic of Korea), and subjected to high-temperature (106.6–123.4 °C) and pressure (1.3 kg/cm^2^) treatment for 16.6–33.4 min using a retort sterilizer (PRS-06-I; Kyunghan Co., Ltd., Gyeongsan, Republic of Korea).

### 4.3. Optimizing Processing Conditions of Fried Fish Cakes Prototype

#### 4.3.1. Optimization of TGase and Sugar Ratios to Improve Texture and Color

The impact of different sugars on the gel strength and chromaticity of fried fish cake prototypes was assessed. Sugars tested included sucrose, glucose, xylose, and trehalose, each at 0.75% concentration. TGase (ACTIVA TGase-K) was added at 0.37%. The prototypes underwent steaming at 55 ± 1 °C for 10 min, followed by oil frying at 160 ± 3 °C for 3 min. The semi-prototypes were cooled to room temperature, vacuum-packed in retort film, and autoclaved at 115 °C for 25 min. Their physical properties were evaluated using a texture analyzer. Yellowness and whiteness measurements were conducted using color difference meter (CM-700d; Konica Minolta Sensing lnc., Tokyo, Japan) to identify the most suitable sugars for the prototype.

#### 4.3.2. Improving Flavor with Various Herbal Oils

To enhance the flavor of the fried fish cake prototypes, various herb oils (bay tree, aniseed, coriander, cumin, and basil oil) were incorporated at 0.1% during the dough mixing process. The intensity of odors in different prototypes was measured using color difference meter (CM-700d; Konica Minolta Sensing lnc., Tokyo, Japan), and sensory evaluations were conducted as described in Section 4.8.

### 4.4. Response Surface Regression Analysis

Optimal conditions for high-temperature and high-pressure processing of the prototypes were determined using dependent variables: DPPH radical scavenging activity (*Y*_1_) for antioxidant activity, whiteness (*Y*_2_) for color change, and gel strength (*Y*_3_) for elasticity. A regression analysis was conducted using the MINITAB statistical software (version 14, MINITAB, State College, PA, USA). The relationships between dependent and independent variables were graphed using MAPLE software (version 12, Maple Soft, Waterloo, ON, Canada). The graph showing the relationship between the independent and dependent variables was expressed as a three-dimensional graph by inputting the values of the constant, first-order terms, second-order terms, and cross-terms, which are the outcomes derived from the regression equation based on the results of the aforementioned regression analysis. Optimal conditions were derived following the methods of Kim et al. [52] and Bezerra et al. [53], utilizing the response optimizer in MINITAB.

### 4.5. Preparation of Controls for Evaluation of Quality Characteristics

To compare the quality characteristics of the final developed fried fish cake prototypes, controls 1 and 2 were prepared using commercial products (Samjin Food Co., Ltd., Busan, Republic of Korea). Controls 1 and 2 included frozen red snapper (74.24%) with additional ingredients like wheat flour, refined salt, xylose, soybean oil, and others. Control 1 was produced without retorting, while control 2 underwent retorting under the same conditions as the developed prototype. Control 3 was prepared identically to the developed prototype but was non-sterilized.

### 4.6. Quality Analysis of Prepared Fried Fish Cake Prototype

#### 4.6.1. Proximate Composition Analysis

The Proximate composition analysis followed the Food Codex method [51]. Carbohydrates were determined by the formula: [100 − (moisture + crude protein + crude fat + ash content)]. Energy was calculated using the FAO/WHO energy conversion factors: [(protein × 4.0) + (fat × 9.0) + (carbohydrate × 4.0)] [54].

#### 4.6.2. pH

The pH value was measured by homogenizing approximately 5 g of the crushed fried fish cake with distilled water (9 times *v*/*w*) using a Homogenizer (SHG-15D; SciLab, Seoul, Republic of Korea). The resulting mixture was then centrifuged at 3000 rpm for 20 min using a centrifuge (Fleta 5; Hanil Scientific Inc., Gimpo, Republic of Korea). The pH was subsequently determined with a pH meter (Ohaus Starter 2100; Ohaus Co., Parsippany, NJ, USA).

#### 4.6.3. Volatile Basic Nitrogen (VBN)

Volatile Basic Nitrogen (VBN) was measured following the method reported in Food Codex [51]. The supernatant obtained from a homogenized mixture was centrifuged at 1100× *g* for 10 min and then filtered through a filter (TY5A-150; Advantec Toyo Kaisha LTD., Tokyo, Japan) and used for VBN measurement, which was calculated using Equation (1).

VBN (mg%) = 0.14 × {(sample titration − control titration) × f}/W × 100 × 50(1)

where f stands for factor of 0.01 N NaOH and W indicates the amount of sample (g).

#### 4.6.4. Volatile Component Intensity (VCI)

Volatile Component Intensity (VCI) was assessed using the method by Kang et al. [55]. In summary, approximately 5 g of fried fish cake sample was placed in a 50 mL conical tube (SPL Life Science Co., Ltd., Gyeonggi, Republic of Korea) and sealed with Parafilm (Heathrow Scientific, Lakeview Parkway, IL, USA) to prevent odor loss. VCI was monitored with an electronic nose (Odor concentration meter, XP-329R; New Cosmos Electric Co., Ltd., Osaka, Japan) set to batch mode, and the odor intensity was expressed in levels.

#### 4.6.5. Measurement of Acid Value

The acid value was determined using the method outlined in the Food Codex [51].

#### 4.6.6. Color Characteristics

The Hunter color values, including lightness (L), redness (a), yellowness (b), and color difference (ΔE) of the fish cake prototype, were recorded with a color difference meter (Konica Minolta Sensing lnc., Tokyo, Japan) following the method by Lee et al. [4]. For reference, the standard white plate had L, a, and b values of 20.69, −4.59, and −9.01, respectively. The color difference (ΔE) was estimated using the formula in Equation (2).

Δ*E* = [(ΔL)^2^ + (Δa)^2^ + (Δb)^2^]^1/2^ (2)

The sample for whiteness measurement was cut into 1 cm × 1 cm (length and width) sections from the inner part of the fried fish cake prototype. After measuring L and b values, the whiteness value was calculated by L − 3b as described by Park [56].

#### 4.6.7. Measurement of Gel Strength

Gel strength was assessed using a texture analyzer (CT3-1000; Brookfield, Middleboro, MA, USA) following the method described by Lee et al. [4]. Briefly, the gel strength of the fried fish cake samples, cut into dimensions of 10 × 10 × 4 mm, was measured using a circular plunger with a 14 mm diameter. The measurement conditions included a distance of 1.5 mm, a test speed of 0.5 mm/s, and a trigger load of 4.0 g.

### 4.7. Antioxidant Activity Analysis

The DPPH radical scavenging activity of the fried fish cake prototypes was calculated following the modified protocol of Teixeira et al. [57]. Five grams sample was mixed with 20 mL of methanol (5-fold dilution) and centrifuged at 3000× *g* for 20 min. The supernatant was filtered using a 0.45 μm pore-sized syringe filter (Hyundai Micro, Seoul, Republic of Korea). All processes were performed at 4 °C or below. The samples were mixed with a 0.2 mM DPPH-radical solution and left to react in the dark for 30 min. The absorbance was then measured at 517 nm using an absorbance meter (SPECTROstar Nano; BMG Labtech, Ortenberg, Hesse, Germany). The DPPH radical scavenging activity was calculated according to the formula of Kim et al. [31].

### 4.8. Sensory Evaluation Analysis

A descriptive evaluation was conducted to select the best herb oil suitable for the fried fish cake prototype using a 7-point rating method by five trained panel members (ages 20–50 years, 2 men and 3 women) affiliated with Samjin Food Co., Ltd. Busan, Republic of Korea. The descriptive analysis items were rated as follows: 7 points for very good, 5 points for average, and 1 point for very bad, evaluating appearance, smell, and overall preference.

The sensory evaluation of the final product prototype was performed using a 9-point scale by a panel of 21 people [8 males (maximum age: 50 years, minimum age: 22 years, average 35.8 ± 8.7) and 13 females (maximum age: 48 years, minimum age: 23 years, average 32.1 ± 7.2)] to evaluate taste, color, odor, texture, and overall acceptance of the final product. The study was conducted according to the guidelines of the Declaration of Helsinki and approved by the Institutional Review Board of Gyeongsang National University [protocol code: GIRB-E24-NY-0010].

### 4.9. Total Amino Acids, Mineral, and Fatty Acid Content

The total amino acids and mineral content of the fried fish cake prototype were measured using the method of the Food Code [51]. Sample oil for fatty acid analysis was extracted following the Bligh and Dyer protocol [58] with chloroform-methanol (2:1, *v*/*v*) used as the extraction solvent. Each analysis was performed with an amino acid autoanalyzer (Pharmacia Biotech Biochrom 30; Biochrom Ltd., Waterbeach Cambridge, UK), inductively coupled plasma optical emission spectrometry (ICP-OES), and gas chromatography (Shimadzu 14A; carrier gas, He; detector, FID) for identification and calculation.

### 4.10. Molecular Weight Analysis

The molecular weight of the proteins in the developed product prototype was determined using Sodium Dodecyl Sulfate Polyacrylamide Gel Electrophoresis (SDS–PAGE; 041BR97428; PowerPac Basic, Bio-Rad Laboratories, Inc., Hercules, CA, USA) following the method by Bashir et al. [59]. Briefly, each lane was loaded with 100 µg protein on precast 4–20% Mini-PROTEAN^®^ TGX™ bis-Tris gels. The electrophoresis was performed in a Tris/glycine/SDS buffer within a Mini-PROTEAN tetra cell at a constant voltage of 100 V. The Precision Plus Protein Dual Color Marker (1610374; Bio-Rad) was used as a standard for molecular weight reference. The gel was stained with Coomassie Brilliant Blue R-250 solution for one hour, followed by destaining for 3 to 4 h. The protein bands were then visualized using the ViewOne Lablite system (EmbiTec, San Diego, CA, USA).

### 4.11. Scanning Electron Microscopy (SEM)

The microstructure of the developed fried fish cake prototype was observed according to the method of Park et al. [60]. Samples were thinly sectioned, freeze-dried, and coated with gold ions using ion sputtering technique (E-1010; Hitachi Instrument Inc., San Jose, CA, USA). The microstructure was then analyzed using a scanning electron microscope (JSM-6490LV; JEOL, Tokyo, Japan). Images were captured with an electron acceleration voltage of 15 kV, and the structure and morphology of the fish cake prototype were examined at low (×100 and ×200) and high (×400) magnifications. Image analysis was conducted using SigmaScan Pro version 5 (Systat Software Inc., San Jose, CA, USA).

### 4.12. Shelf-Life Analysis

The expiration date of the developed prototype was predicted using the Visual Shelf-Life Simulator for Foods (VSLSF), a food expiration date calculation system provided by the Food Codex [51]. To accurately predict the expiration date, an accelerated test was conducted at three storage temperatures (25, 35, and 45 °C). Quality indicators such as general bacterial count, *Escherichia coli*, acid value, and sensory test results were input into the VSLSF. The consumption period of the prototype was determined by multiplying the predicted value by a safety factor of 0.8.

### 4.13. Statistical Analysis

All experimental analyses were performed in triplicate. The results are expressed as means ± standard deviation (S.D.). Statistical analyses were conducted using ANOVA in SPSS Statistics version 18 (IBM Corp., Armonk, NY, USA). Post hoc comparisons were made using Duncan’s multiple range test, with statistical significance set at *p* < 0.05.

## Figures and Tables

**Table 1:** Gel strength of the prototype according to the addition of TGase and the presence or absence of heating (unit: g × cm).

Category	Gel Strength (g × cm)	
	No TG Added	TG Added
No Heating	111.17 ± 4.76 ^a^	111.10 ± 4.76 ^a^
Heating	132.53 ± 3.87 ^b^	156.52 ± 5.62 ^b^

Values are means ± S.D. of three replications (*n* = 3). Different superscript letters (a, b) indicate significant differences among means by Duncan’s test (*p* < 0.05).

**Table 2:** Gel strength, yellowness, and whiteness of fried fish cake samples with different added sugars.

Parameter	Sucrose	Glucose	Xylose	Trehalose
Gel strength (g × cm)	163.20 ± 3.56 ^b^	150.33 ± 5.10 ^a^	177.66 ± 6.62 ^c^	187.34 ± 9.82 ^c^
Yellowness	6.67 ± 0.10 ^a^	13.03 ± 0.49 ^b^	26.05 ± 0.69 ^c^	6.53 ± 0.47 ^a^
Whiteness	49.41 ± 0.57 ^d^	28.57 ± 0.39 ^b^	−31.06 ± 1.55 ^a^	47.29 ± 0.67 ^c^

Values are means ± S.D. of three replications (*n* = 3). Different superscript letters (a–d) indicate significant differences among means by Duncan’s test (*p* < 0.05).

**Table 3:** Odor intensity and sensory scores of fried fish cake with herbal oil addition.

Sample	Odor Intensity (Level)	Sensory Score (Point)
Control group	391.3 ± 13.6 ^a^	6.4 ± 0.1 ^c^
Tocopherol	388.6 ± 19.4 ^a^	7.6 ± 0.3 ^d^
Bay leaf	468.8 ± 22.1 ^b^	7.4 ± 0.2 ^d^
Coriander	521.9 ± 20.5 ^c^	4.8 ± 0.2 ^a^
Cumin	499.3 ± 17.3 ^b, c^	5.5 ± 0.4 ^b^
Basil	512.9 ± 25.5 ^c^	5.2 ± 0.3 ^a, b^
Anisid	411.0 ± 36.1 ^a^	5.6 ± 0.1 ^b^

Values are means ± S.D. of three replications (*n* = 3). Different superscript letters (a–d) indicate significant differences among means by Duncan’s test (*p* < 0.05).

**Table 4:** Estimated coefficients and *p* values of the second-order regression equation for optimization of manufacturing conditions for room-temperature fried fish cake prototype.

Variable	*Y*_1_		*Y*_2_		*Y*_3_	
	Coefficient	*p* Value	Coefficient	*p* Value	Coefficient	*p* Value
Constant	63.197	0.000	53.357	0.000	236.807	0.000
*X*_1_	4.311	0.000	0.061	0.847	0.263	0.708
*X*_2_	−3.975	0.000	−3.809	0.000	−15.945	0.000
*X*_3_	−1.414	0.025	−1.108	0.008	−12.640	0.000
*X*_1_ *X*_1_	−0.954	0.124	−0.376	0.301	−0.097	0.900
*X_2_X_2_*	−1.626	0.021	0.473	0.203	−4.233	0.001
*X*_3_ *X*_3_	−1.290	0.050	−0.446	0.227	−2.254	0.019
*X*_1_ *X*_2_	0.175	0.795	0.063	0.880	−0.113	0.902
*X*_1_ *X*_3_	−0.375	0.581	−0.213	0.611	−0.287	0.753
*X*_2_ *X*_3_	0.025	0.970	1.938	0.002	−1.038	0.277

*X*_1_: Amount of antioxidant added (%); *X*_2_: Temperature conditions during the high-temperature and high-pressure treatment process (°C); *X*_3_: Time conditions in the high-temperature and high-pressure treatment process (min); *Y*_1_: DPPH (%); *Y*_2_: Whiteness; *Y*_3_: Gel strength (g × cm); *X*_1_*X*_1_, *X*_2_*X*_2_, *X_3_X*_3_, *X*_1_*X*_2_, *X*_1_*X*_3_, and *X*_2_*X*_3_: Coefficients of the quadratic regression equation.

**Table 5:** Optimal conditions predicted for the production of room-temperature fried fish cake prototype.

Independent Variable	Value	*X*_1_		*X*_2_		*X*_3_	
*Y*_1_	TV	70.0	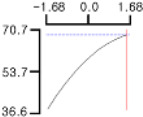	70.0	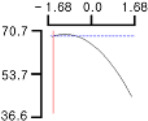	70.0	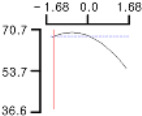
	CV	1.68		−1.63		−1.62	
	AC	0.18		106.9		16.9	
*Y*_2_	TV	53.0	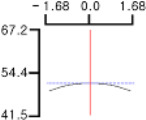	53.0	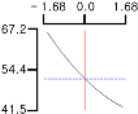	53.0	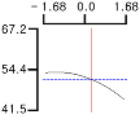
	CV	0.00		0.00		0.29	
	AC	0.10		115.0		30.5	
*Y*_3_	TV	230.0	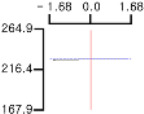	230.0	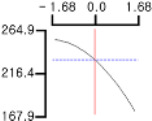	230.0	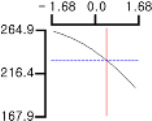
	CV	0.00		0.00		0.49	
	AC	0.10		115.0		27.5	
Optimization conditions for all dependent variables	CV	1.08		−0.49		0.75	
	AC	0.15%		112.6 °C		28.8 min	

*X*_1_: Amount of antioxidant added (%); *X*_2_: Temperature conditions during the high-temperature and high-pressure treatment process (°C); *X*_3_: Time conditions in the high-temperature and high-pressure treatment process (min); *Y*_1_: DPPH (%); *Y*_2_: Whiteness; *Y*_3_: Gel strength (g × cm); TV: Target value; CV: Coded value; AC: Actual experimental value.

**Table 6:** Predicted value and experimental value of various dependent variables derived under optimal conditions.

Dependent Variable	Predicted Value	Experimental Value
*Y*_1_ (DPPH, %)	66.1 ^a^	65.2 ± 1.7 ^a^
*Y*_2_ (Whiteness)	52.9 ^a^	52.8 ± 0.9 ^a^
*Y*_3_ (Gel strength, g × cm)	233.2 ^a^	235.1 ± 5.9 ^a^

Values are means ± S.D. of three replications (*n* = 3). *Y*_1_: DPPH (%); *Y*_2_: Whiteness; *Y*_3_: Gel strength (g × cm). Different superscript letters (a) indicate significant differences among means by Duncan’s test (*p* < 0.05).

**Table 7:** Proximate composition, pH, and energy of the controls and the developed fried fish cake prototype.

Sample	Proximate Components (g/100 g)					pH	Energy (kcal/100 g)
	Moisture	Protein	Fat	Ash	Carbohydrate		
Control 1	57.3	12.5	2.3	3.4	24.5	6.6	168.7
Control 2	56.8	13.0	2.2	4.1	23.9	6.2	167.4
Control 3	71.9	10.5	1.3	1.9	14.4	6.9	111.3
Fried fish cake prototype	72.4	10.3	1.4	1.9	14.0	6.9	109.8

Ash = 100 − (Moisture + Protein + Fat + Carbohydrates); Energy = [(Protein × 4.0) + (Fat × 9.0) + (Carbohydrates × 4.0)].

**Table 8:** Total amino acid content of control and the developed fried fish cake prototype.

Amino Acids	RDR (g/70 kg Body Weight)	Control 1	Control 2	Control 3	FFCP
Histidine	1.6	0.31 (2.5)	0.26 (2.3)	0.20 (2.1)	0.23 (2.3)
Isoleucine	3	0.56 (4.5)	0.50 (4.4)	0.44 (4.7)	0.52 (5.1)
Leucine	6.1	1.03 (8.3)	0.94 (8.3)	0.81 (8.6)	0.90 (8.9)
Lysine	4.8	1.04 (8.4)	0.94 (8.3)	0.95 (10.1)	0.96 (9.4)
SAA	2.3	0.53 (4.3)	0.52 (4.6)	0.42 (4.5)	0.50 (4.9)
AAA	4.1	0.86 (7.0)	0.80 (7.0)	0.70 (7.4)	0.80 (7.8)
Threonine	2.5	0.56 (4.5)	0.52 (4.6)	0.45 (4.8)	0.48 (4.7)
Valine	4	0.6 (4.8)	0.56 (4.9)	0.46 (4.9)	0.54 (5.3)
∑EAA	28.4	5.49 (44.3)	5.04 (44.4)	4.43 (47.1)	4.93 (48.4)
Aspartic acid		1.14 (9.2)	1.05 (9.2)	0.97 (10.3)	1.09 (10.7)
Serine		0.53 (4.3)	0.49 (4.3)	0.42 (4.4)	0.42 (4.1)
Glutamic acid		2.82 (22.8)	2.59 (22.8)	1.78 (18.8)	1.66 (16.3)
Proline		0.60 (4.8)	0.55 (4.8)	0.29 (3.1)	0.34 (3.3)
Glycine		0.48 (3.9)	0.45 (4.0)	0.42 (4.4)	0.46 (4.5)
Alanine		0.67 (5.3)	0.61 (5.4)	0.54 (5.7)	0.61 (6.0)
Arginine		0.70 (5.6)	0.60 (5.3)	0.60 (6.4)	0.66 (6.5)
∑NAA		6.94 (55.9)	6.34 (55.8)	5.02 (53.1)	5.24 (51.4)

RDR: Recommended Daily Requirement according to Protein and Amino Acid Requirements in Human Nutrition [41]. FFCP: Fried fish cake prototype; SAA: Sulfur amino acids (cysteine, methionine). AAA: Aromatic amino acids (tyrosine, phenylalanine). ∑EAA: Sum of essential amino acids. ∑NAA: Sum of nonessential amino acids.

**Table 9:** Mineral content of controls and the developed fried fish cake prototype.

Mineral (mg/100 g)	Samples			
	Control 1	Control 2	Control 3	FFCP
Ca	463.5	470.5	33.4	35.3
P	192.5	196.9	106.9	116.0
K	139.8	139.4	65.6	66.4
Fe	0.75	0.73	0.10	0.05
Mg	21.6	21.6	13.5	14.1

Ca: Calcium; P: Phosphorus; K: Potassium; Fe: Iron; Mg: Magnesium; FFCP: Fried Fish Cake Prototype.

**Table 10:** Fatty acid content in the controls and the developed fried fish cake prototype.

Fatty Acids (g/100 g)		Samples			
		Control 1	Control 2	Control 3	FFCP
C4:0	Butyric acid	-	-	-	-
C6:0	Caproic acid	-	-	-	-
C8:0	Caprylic acid	-	-	-	-
C10:0	Capric acid	-	-	-	-
C12:0	Lauric acid	-	-	-	-
C13:0	Tridecanoic acid	-	-	-	-
C14:0	Myristic acid	0.02	0.02	0.01	0.01
C15:0	Pentadecanoic acid	-	0.01	-	-
C15:1	Pentadecenoic acid	-	-	-	-
C16:0	Palmitic acid	0.33	0.35	0.17	0.15
C17:0	Margaric acid	0.01	0.01	-	-
C18:0	Stearic acid	0.10	0.11	0.06	0.05
C20:0	Arachidic acid	0.01	0.01	-	-
C21:0	Heneicosanoic acid	-	-	-	-
C22:0	Behenic acid	0.01	0.01	0.01	-
C23:0	Tricosanoic acid	-	-	-	-
C24:0	Lignoceric acid	-	-	-	-
C14:1n-5	Myristoleic acid	-	-	-	-
C16:1n-7	Palmitoleic acid	0.02	0.02	-	0.01
C17:1	Margaroleic acid	-	-	-	-
C18:1t	Elaidic acid	-	-	-	0.01
C18:1c	Oleic acid	0.45	0.40	0.28	0.26
C20:1n-9	cis-11-Eicosenoic acid	0.01	0.01	0.01	0.01
C18:2t	Linolelaidic acid	-	-	-	-
C18:2c	Linoleic acid	0.95	0.84	0.53	0.36
C18:3γ	γ-Linolenic acid	0.01	0.01	0.01	-
C18:3n-3	α-Linolenic acid	0.08	0.07	0.05	0.03
C20:2n-6	cis-11,14-Eicosadienoic acid	-	-	-	-
C20:3n-6	dihomo γ-Linolenic acid	-	-	-	-
C20:3n-3	cis-11,14,17-Eicosatrienoic acid	-	-	-	-
C20:4n-6	Arachidonic acid	0.02	0.03	-	0.01
C20:5n-3	Eicosapentaenoic acid	0.03	0.04	0.04	0.04
C22:1n-9	Erucic acid	0.01	0.01	0.01	-
C22:2n-6	Docosadienoic acid	-	-	-	-
C22:6n-3	Docosahexaenoic acid	0.12	0.16	0.08	0.09
C24:1n-9	Nervonic acid	-	-	-	-
Total fatty acids		2.18	2.11	1.26	1.03

-: Not Detected; FFCP: Fried fish cake prototype.

**Table 11:** Odor intensity, volatile basic nitrogen, acid value, and Hunter color value of control and the developed fried fish cake product.

Experimental Items	Control 1	Control 2	Control 3	FFCP
Odor intensity (level)	864.8 ± 8.62 ^b^	969.3 ± 7.56 ^c^	535.4 ± 6.26 ^a^	544.11 ± 14.35 ^a^
VBN (mg/100 g)	2.8	3.1	1.0	1.1
Acid value (mg/100 g)	14.7	21.3	0.2	0.0
L value	47.03 ± 3.92 ^a^	58.21 ± 2.24 ^b^	77.50 ± 0.65 ^c^	76.47 ± 0.53 ^c^
a value	0.35 ± 0.06 ^b^	10.96 ± 0.87 ^c^	−2.76 ± 0.05 ^a^	−2.29 ± 0.02 ^a^
b value	15.32 ± 0.89 ^b^	30.48 ± 0.85 ^c^	6.81 ± 0.07 ^a^	7.55 ± 0.09 ^a^
Whiteness	21.08 ± 3.15 ^b^	−33.24 ± 3.40 ^a^	57.06 ± 0.46 ^c^	53.83 ± 0.31 ^c^
ΔE value	52.56 ± 1.52 ^a^	56.65 ± 2.25 ^a, b^	59.00 ± 3.78 ^b^	58.23 ± 1.66 ^b^
Gel strength (g × cm)	231.80 ± 12.79 ^a^	256.93 ± 3.74 ^b^	244.40 ± 6.92 ^a, b^	279.26 ± 15.42 ^c^

Values are means ± S.D. of three replications (*n* = 3). L: Lightness; a: Redness; b: Yellowness; ΔE: Color difference; VBN: Volatile basic nitrogen; FFCP: Fried fish cake prototype. Different superscript letters (a–c) in each row indicate significant differences among means by Duncan’s test (*p* < 0.05).

**Table 12:** Sensory evaluation of control and the developed fried fish cake prototype using a 7-point scale method.

Sample	Sensory Evaluation (Score)				
	Taste	Color	Odor	Texture	Overall Acceptance
Control 1	5.0 ± 0.18 ^b^	4.6 ± 0.20 ^b^	4.5 ± 0.23 ^b^	5.3 ± 0.17 ^b^	5.2 ± 0.14 ^b^
Control 2	4.4 ± 0.21 ^a^	4.0 ± 0.24 ^a^	3.5 ± 0.25 ^a^	4.5 ± 0.10 ^a^	4.8 ± 0.11 ^a^
Control 3	6.8 ± 0.15 ^c^	6.5 ± 0.15 ^c^	6.2 ± 0.15 ^c^	6.8 ± 0.12 ^c^	6.7 ± 0.12 ^c^
FFCP	6.8 ± 0.22 ^c^	6.6 ± 0.13 ^c^	6.3 ± 0.16 ^c^	6.7 ± 0.11 ^c^	6.7 ± 0.15 ^c^

Values are means ± S.D. of three replications (*n* = 3). FFCP: Fried fish cake prototype. Different superscript letters (a–c) in each column indicate significant differences among means by Duncan’s test (*p* < 0.05).

**Table 13:** Acid value (mg KOH/g) and sensory evaluation of the developed fried fish cake prototype stored at room-temperature for shelf-life prediction.

Storage Temperature	Day	Acid Value and Sensory Evaluation					
		Acid Value	Color	Odor	Taste	Texture	Overall Acceptance
25 °C	0	1.2 ± 0.1 ^a^	7.0 ± 0.5 ^e, f^	7.0 ± 0.5 ^b, c^	7.0 ± 0.5 ^c, d^	7.0 ± 0.5 ^b, c^	7.0 ± 0.4 ^c, d^
	30	2.7 ± 0.0 ^b^	6.5 ± 0.0 ^e^	6.8 ± 0.5 ^b, c^	6.5 ± 0.0 ^c^	6.5 ± 0.0 ^b^	6.6 ± 0.2 ^b, c^
	60	5.0 ± 0.0 ^c^	6.3 ± 0.0 ^d^	6.8 ± 0.5 ^b, c^	6.5 ± 0.0 ^c^	6.5 ± 0.0 ^b^	6.6 ± 0.1 ^b^
	90	5.4 ± 0.2 ^d^	6.2 ± 0.0 ^c^	6.2 ± 0.5 ^a, b^	6.5 ± 0.0 ^c^	6.3 ± 0.4 ^a, b^	6.4 ± 0.2 ^b^
	120	6.2 ± 0.2 ^e^	5.7 ± 0.4 ^a, b^	5.8 ± 0.4 ^a^	6.2 ± 0.0 ^b^	6.3 ± 0.4 ^a, b^	6.2 ± 0.4 ^a, b^
	150	6.2 ± 0.3 ^e, f^	5.6 ± 0.2 ^a^	5.8 ± 0.2 ^a^	6.0 ± 0.2 ^a^	6.1 ± 0.3 ^a^	6.1 ± 0.3 ^a^
35 °C	0	1.2 ± 0.1 ^a^	7.0 ± 0.5 ^c, d^	7.0 ± 0.5 ^c, d^	7.0 ± 0.5 ^c, d^	7.0 ± 0.5 ^b, c^	7.0 ± 0.4 ^b, c^
	30	1.9 ± 0.1 ^b^	6.5 ± 0.0 ^b, c^	6.6 ± 0.4 ^c, d^	6.8 ± 0.5 ^c, d^	6.8 ± 0.5 ^b, c^	6.7 ± 0.3 ^b, c^
	60	4.9 ± 0.1 ^c^	6.3 ± 0.4 ^b, c^	6.0 ± 0.5 ^b, c^	6.5 ± 0.0 ^c^	6.5 ± 0.0 ^b^	6.3 ± 0.1 ^b^
	90	8.4 ± 0.3 ^d^	6.2 ± 0.5 ^b, c^	5.7 ± 0.0 ^b^	6.0 ± 0.5 ^b, c^	6.2 ± 0.5 ^a, b^	6.0 ± 0.1 ^a, b^
	120	9.4 ± 0.3 ^e^	5.7 ± 0.0 ^b^	5.0 ± 0.4 ^a^	5.7 ± 0.0 ^b^	5.7 ± 0.0 ^a^	5.5 ± 0.1 ^a^
	150	10.7 ± 0.2 ^f^	5.4 ± 0.1 ^a^	4.9 ± 0.2 ^a^	5.5 ± 0.1 ^a^	5.6 ± 0.1 ^a^	5.5 ± 0.2 ^a^
45 °C	0	1.2 ± 0.1 ^a^	7.0 ± 0.5 ^c, d^	7.0 ± 0.5 ^c, d^	7.0 ± 0.5 ^c, d^	7.0 ± 0.5 ^c, d^	7.0 ± 0.4 ^c, d^
	30	6.0 ± 0.1 ^b^	6.5 ± 0.0 ^b, c^	6.5 ± 0.0 ^c^	6.5 ± 0.0 ^c^	6.6 ± 0.4 ^c^	6.6 ± 0.1 ^c^
	60	6.7 ± 0.1 ^c^	6.3 ± 0.4 ^b, c^	5.8 ± 0.4 ^a, b^	5.8 ± 0.4 ^b, c^	6.0 ± 0.5 ^b, c^	6.0 ± 0.1 ^b, c^
	90	9.6 ± 0.4 ^d^	6.2 ± 0.5 ^b, c^	5.2 ± 0.5 ^a^	5.5 ± 0.4 ^a, b^	5.7 ± 0.0 ^b^	5.7 ± 0.2 ^b^
	120	11.4 ± 0.4 ^e^	5.7 ± 0.0 ^b^	5.0 ± 0.4 ^a^	5.2 ± 0.5 ^a^	5.5 ± 0.4 ^a, b^	5.3 ± 0.1 ^a^
	150	13.2 ± 0.2 ^f^	5.3 ± 0.1 ^a^	4.8 ± 0.2 ^a^	4.9 ± 0.2 ^a^	5.3 ± 0.3 ^a^	5.2 ± 0.2 ^a^

Values are mean ± S.D. Different letters (a–f) in each column at each temperature indicate significant differences among means by Duncan’s test (*p* < 0.05).

**Table 14:** Shelf-life estimation using accelerated testing (utilizing the Shelf-Life Prediction program provided by the Korean MFDS) [51].

Determined Parameter	Response Order	Temperature (°C)	*R* ^2^	Predicted Shelf-Life (Month)
Acid value	Zero order	25	0.9583	23.93
		35	0.9377	
		45	0.9782	
	First order	25	0.9040	9.70
		35	0.9032	
		45	0.8489	

MFDS: Korean Ministry of Food and Drug Safety; *R*^2^: Coefficient of determination.

## Data Availability

The original contributions presented in the study are included in the article, further inquiries can be directed to the corresponding authors.
